# Hierarchical climate-driven dynamics of the active channel length in temporary streams

**DOI:** 10.1038/s41598-021-00922-2

**Published:** 2021-11-02

**Authors:** Gianluca Botter, Filippo Vingiani, Alfonso Senatore, Carrie Jensen, Markus Weiler, Kevin McGuire, Giuseppe Mendicino, Nicola Durighetto

**Affiliations:** 1 Department of Civil, Environmental and Architectural Engineering, University of Padua, Via Marzolo 9, 35131 Padua, PD Italy; 2 Department of Environmental Engineering, University of Calabria, Via Pietro Bucci 42, 87036 Arcavacata di Rende, CS Italy; 3 Department of Forest Resources and Environmental Conservation, Virginia Tech, Cheatham Hall, 210B, 310 West Campus Drive, Blacksburg, VA 24061 USA; 4 Fakultät für Umwelt und Natürliche Ressourcen, Universität Freiburg, Friedrichstr. 39, 79098 Freiburg, Germany

**Keywords:** Geomorphology, Hydrology

## Abstract

Looking across a landscape, river networks appear deceptively static. However, flowing streams expand and contract following ever-changing hydrological conditions of the surrounding environment. Despite the ecological and biogeochemical value of rivers with discontinuous flow, deciphering the temporary nature of streams and quantifying their extent remains challenging. Using a unique observational dataset spanning diverse geomorphoclimatic settings, we demonstrate the existence of a general hierarchical structuring of river network dynamics. Specifically, temporary stream activation follows a fixed and repeatable sequence, in which the least persistent sections activate only when the most persistent ones are already flowing. This hierarchical phenomenon not only facilitates monitoring activities, but enables the development of a general mathematical framework that elucidates how climate drives temporal variations in the active stream length. As the climate gets drier, the average fraction of the flowing network decreases while its relative variability increases. Our study provides a novel conceptual basis for characterizing temporary streams and quantifying their ecological and biogeochemical impacts.

## Introduction

River networks shape the Earth landscape transporting water and sediments from the uplands to the sea, and provide a number of invaluable ecosystem services [e.g.]^1,2^. For decades, the spatial organization of streams has stimulated scientific debate around observed morphological and ecological patterns in river basins. At fine scales, the location of the channel heads has been the focus of pioneering work^3,4^ that fuelled an array of methodologies for the analysis of Digital Terrain Maps; at larger spatial scales, theoretical investigations examined origins and implications of the branching shape of river networks and their fractal nature^5–10^. Recently, the temporal dimension of river networks has been recognized as a core issue not only over geological timescales typical of landscape evolution models, but also during individual years or seasons, within which river segments may temporarily cease to flow [e.g.]^11–13^. This empirical evidence has shown that the active portion of channel networks experiences a continuous sequence of expansion and contraction cycles driven by precipitation. Temporary streams, here defined as fluvial systems that periodically cease to flow, are observed in a wide range of climatic settings and represent an important fraction of global rivers^11,14^. The study of active stream dynamics is pivotal not only to characterize spatial patterns of hydrologic regimes but also to understand the influence of flow intermittency on e.g. ecological dispersion, in-stream processes, hyporheic exchange and nutrient spiraling^15–20^.

Growing empirical efforts in monitoring network dynamics have also provided enhanced insight into the underlying driving processes. In particular, recent work has suggested that the transitions between dry and wet channels are governed by the local imbalance between the capacity to transmit flow in the subsurface and the seepage flow delivered from upslope—usually conceived as proportional to the local contributing area^12,21^. As the local transmissivity and the contributing area may be durable features of a landscape, this conceptual scheme may also suggest the existence of a hierarchy in the activation of different branches of temporary streams as a function of the underlying catchment wetness. However, the activation order of different reaches of a dynamic stream network has never been quantitatively explored in the literature, and the potential implications for the dynamics of the active drainage density under diverse hydroclimatic conditions have not been disclosed. Furthermore, a comparative analysis of stream length variability across different climatic regimes is lacking.

Here a combination of theoretical analysis and empirical observations is used to address the following questions:

- Is there a mathematical relationship between the activation or deactivation order of different reaches of a dynamic network and the corresponding degree of flow persistency of those reaches? And what are the implications in terms of network spatial correlation and temporal changes of active length?
- Can we identify constraints in the temporal variations of the active drainage density that incorporate the complex effect of climate?

Due to the unique contribution of temporary streams to biodiversity and nutrient cycling [e.g.]^22,23^, these issues are becoming urgent for identifying sustainable water management strategies and assessing the full ecological and biogeochemical function of drainage networks^19,24–26^.

**Figure 1:**
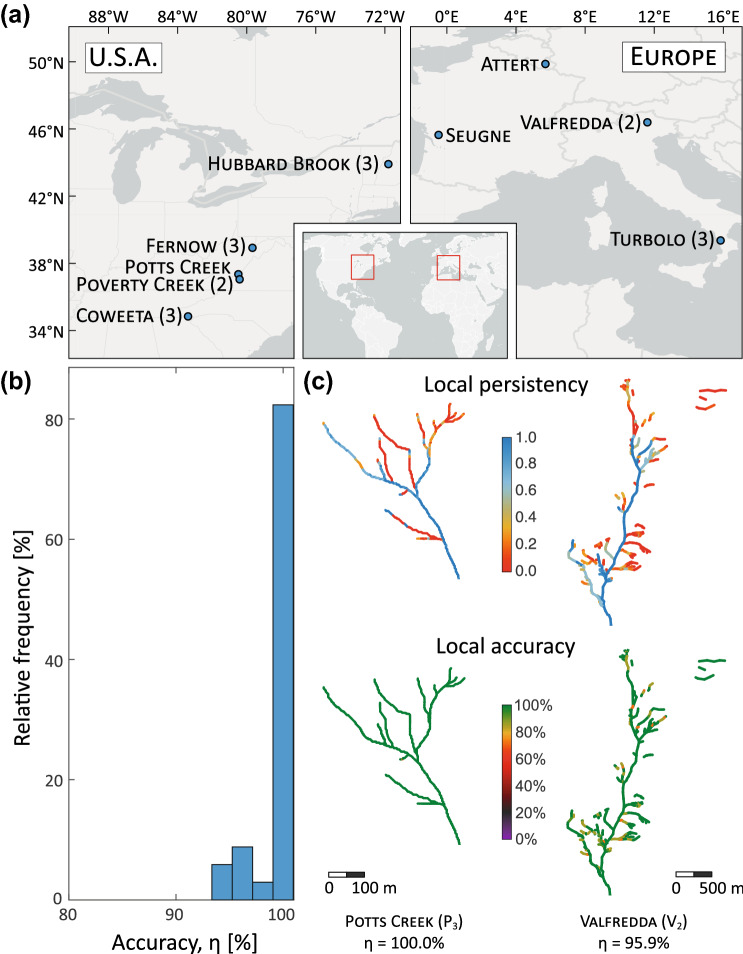
Study sites and performance of the hierarchical model, in which the activation (deactivation) of the nodes follows a strictly decreasing (increasing) order of persistency. (**a**) Geographical location of the study catchments across Europe and eastern USA. The number of case studies for each location is indicated within round brackets only when this number is larger than one. Map generated with Arcgis Pro 2.7.1 (www.arcgis.com). The histogram in (**b**) represents the relative frequency of the accuracy of the hierarchical model (relative number of nodes properly classified as wet or dry by the hierarchical model) for all the study catchments. The maps in (**c**) show spatial patterns of local persistency and local accuracy of the hierarchical model in the best and the worst cases (respectively South Fork of Potts Creek, USA and Valfredda Creek, Italy). Analogous maps for all the study sites are reported in Figures S2 and S5 of the Supplementary Information. For comparison, the corresponding maps of local accuracy obtained from the random model are represented in Figure S6.

The basic hypothesis of this work is that the local persistency of different river stretches of a stream network (i.e. the percentage of time during which water is flowing over different locations of a watercourse) defines their activation and deactivation order during individual storm events and across seasons. We also postulate that the mean persistency of the entire network represents the primary link between the temporal variability of active stream length and the underlying climatic drivers. While intuitive, these hypotheses have never been formally assessed in the existing literature and—most importantly—their implications in terms of active length dynamics have not been disclosed. Here, the proposed research hypotheses were tested analyzing seasonal (or annual) event-based dynamics of the active network in 19 European and North-American catchments (Fig. 1a), encompassing a broad range of climatic conditions, land-cover, drainage areas and geology.

## Hierarchical activation of temporary streams

For each study site, flow presence maps representing the observed active network on different dates were produced using multi-month field mapping campaigns (with a mean frequency of two weeks) or water presence sensors (with a sub-hourly resolution). These maps were converted into sets of geo-referenced nodes with binary states (wet/dry), and the local persistency of each node ($P_i$) was calculated as the relative fraction of data indicating the presence of flowing water in that location. Afterwards, each flow presence map was compared with the outcome of a hierarchical model in which the network nodes were switched on from the most to the least persistent, until the total number of nodes observed as active during the corresponding survey was reached (Methods). Regardless of the underlying climatic and geologic complexity, the accuracy of the hierarchical model (here defined as the relative number of nodes properly classified as wet or dry throughout a river network during all the surveys) typically exceeded 99% (Fig. 1b), with a minimum accuracy around $95\%$ for two European rivers, the Attert and the Valfredda (Fig. 1c). The average accuracy is very high, and much higher than the accuracy of a random model that assigns the status of each node at random independently on the status of the other nodes but consistently with the local persistency, which is $0.79 \pm 0.075\%$ for our selection of case studies (SI). Thus, our observations suggest that the most persistent nodes were generally the first to activate during individual rainfall events and the last to dry-out during the subsequent recessions. The less persistent nodes, instead, were mostly switched on only in response to the most significant rain events and when all the nodes with a higher persistency were already active. This behaviour is aligned with that foreseen by the conceptual model proposed by^12,21^, based on a set of physical assumptions which are not required by the purely statistical approach developed in this paper.

The most common exceptions to the hierarchical behaviour observed in our set of case studies were the following: (1) nodes with a relatively low persitency that activate in the early stage of a rain event (Poverty Creek); (2) lack of symmetry in the rate and/or the direction of wetting and drying processes (Valfredda, Turbolo); (3) inter-event shifts in the wetting mechanism (e.g. from the downstream propagation of a saturation front induced by intense events taking place after dry periods to the upstream expansion of saturated areas around the permanent network, as observed in the Valfredda). In spite of this complexity, the hierarchical phenomenon was dominant across the full range of climatic conditions, temporal resolutions and spatial scales explored in the paper. In particular, the hierarchical activation rule applied also to disconnected river networks, where the nodes with the same persistency are not necessarily adjacent because of local discontinuities of landscape and geology (see Fig. 1c). This means that the hierarchical phenomenon is much more than a simple upstream/downstream propagation of a saturation front along the network^12^. In particular, our results indicate that stream sections potentially quite distant in the physical space (up to tens or hundreds of kilometers) might share the same average degree of persistency and exhibit systematically synchronous dynamics. Furthermore, the hierarchical scheme held also within catchments as large as several hundreds of $\mathrm{km}^2$ (the Attert and the Seugne). These basins are characterized by significant internal landscape and climatic gradients that are potentially responsible for the unsystematic, selective activation of limited and specific portions of the river network. The ability of the hierarchical model to describe active stream dynamics in these larger river basins indicates that meso-scale channel network dynamics maximize the spatial correlation of the nodes’ states (see Methods) and reflect a homogeneous signal, such as the total catchment storage, driven by mid-range antecedent precipitation (e.g. weekly to monthly rainfall, see^27^). Interestingly, the spatial homogeneity of the hydrological signal underpinning the hierarchical dynamics of temporary streams promotes the synchronization of node status within a channel network, regardless of the observed complex patterns of local persistency produced by spatially heterogeneous physiographic and morphometric features (see Fig. 1c and^28^).

The results shown in Fig. 1 bear important practical and theoretical implications. For instance, the hierarchical behaviour of temporary streams can streamline empirical surveys of river network dynamics, with beneficial consequences on the ability to characterize spatiotemporal patterns of flow persistency^29,30^. In perfectly hierarchical networks, the adoption of ad-hoc monitoring strategies that exploit information available from previous surveys can reduce the number of nodes to monitor during a long-term field campaign with at least 10 surveys by 40 to 75%, depending on the exact number of surveys and the mean network persistency (Supplementary Information). Although several initial surveys that include most of the network are needed to establish the hierarchy among the nodes, the saving in terms of number of surveys becomes particularly significant when the number of surveys exceeds 20—as typically needed to capture event-based variations of the active length, see Table 1 in^31^. This indicates that the hierarchical nature of stream dynamics could significantly facilitate the observational mapping of flowing streams in a broad range of settings, especially in large river basins. Furthermore, the existence of a fixed hierarchical order in the expansion and retraction of temporary rivers allows the identification of a one-to-one relationship between the total length of flowing streams and the spatial distribution of active nodes in the network, if a spatial map of the local persistency is known e.g. based on multiple field surveys. This could help the reconstruction and the modeling of channel network dynamics, especially in cases where active length statistics can be indirectly inferred from rainfall and/or discharge observations^12,27,28,32^.

## Quantifying the mean active drainage density and its temporal variations

The observed hierarchical behaviour of river networks enabled the derivation of novel theoretical results that advance our ability to predict the dynamics of flowing channels in temporary streams. In hierarchical networks, in fact, active channel length statistics can be expressed analytically in terms of the mean network persistency, unveiling the complex linkage between climate and stream dynamics.

A river network was described by *N* nodes with a random binary state (wet/dry). The status of each node represents the hydrologic conditions of the portion of network that is associated to that specific node. To avoid the issue of identifying the status of a link connecting two neighbouring nodes with different states (wet/dry), the reach associated to the node *i* ($i \in (1,N)$) is not a link with one of the adjacent nodes but rather a stretch embedding the node *i* (Methods). Shifts in the status of the nodes produce changes in the flowing stream length, which are here characterized by the temporal variations of the active drainage density, *D* (defined as the active stream length normalized with the corresponding catchment area,^33^). In this framework, the average drainage density, ${\bar{D}}$, can be expressed as (Methods):

1 $$\begin{aligned} {\bar{D}} = {\bar{P}} D_g \, , \end{aligned}$$

where ${\bar{P}}$ is the mean node persistency throughout the entire network (${\bar{P}}= \sum _{i=1}^N P_i / N$ in case of equally spaced nodes) and $D_g$ is the potential geomorphic drainage density, that corresponds to the simultaneous activation of all the *N* nodes in the channelized network, including streams that are typically dry but excluding what is out of the geomorphically obvious channel features and the hillslopes (where surface flow could be temporarily observed). When the activation of the nodes is hierarchical, the temporal coefficient of variation of the active drainage density, $CV_D$ (that also corresponds to the temporal coefficient of variation of the active network length, $CV_L$) can be written as (Methods):

2 $$\begin{aligned} CV_D = CV_L = \sqrt{ \frac{{\bar{P}}(1-{\bar{P}}) + \widetilde{\Delta P}}{{\bar{P}}^2} } \, , \end{aligned}$$

where the term $\widetilde{\Delta P} = 2 / N^2 \cdot \sum _{i=1}^N i (P_i- {\bar{P}}) \le 0$ represents a weighted spatial average of the persistency deviations around the mean. Thus, $\widetilde{\Delta P}$ reflects spatial variation of persistency likely induced by patterns of geologic and morphometric attributes in the channel network or landscape.

Equation (2) allows for the identification of two distinct contributions to the temporal changes of the drainage density: a positive contribution proportional to ${\bar{P}}(1-{\bar{P}})$, determined by the mean persistency of the whole stream network, and a negative contribution proportional to $\widetilde{\Delta P}$, that depends on the internal spatial variability of *P*. Thus, an upper limit for the coefficient of variation of the active drainage density can be identified by setting $\widetilde{\Delta P} = 0$ in Eq. (2):

3 $$\begin{aligned} CV_D^{max} = CV_L^{max} = \sqrt{\frac{1-{\bar{P}}}{{\bar{P}}}}\, . \end{aligned}$$

Equation (3) corresponds to the theoretical coefficient of variation of a homogeneous network made up of synchronous nodes sharing the same persistency (${\bar{P}}$). In realistic cases, instead, the actual value of $CV_D$ is influenced by the spatial variations of the local persistency. Enhanced internal geological, landscape and hydrological heterogeneities entail the spatial changes of flow persistence and increase $| \widetilde{\Delta P} |$, thereby reducing the variability of the active length. An emblematic example of this trend is represented by the catchments $H_2$ (Hubbard Brook, USA) and $V_1$ (Valfredda, Italian Alps). These are two case studies with a different degree of landscape heterogeneity that share the same mean persistency (${\bar{P}} \simeq 0.6$). As predicted by Eq. (2), the observed value of $CV_D$ in $V_1$, whose contributing catchment is characterized by five distinct lithological types and four diverse land uses^27^, is approximately $40\%$ lower than that of $H_2$, where vegetation and geology are more homogeneous in comparison^28^.

To describe the influence of the spatial variability of the local persistency on $CV_D$, we developed an analytical model, in which a one-parameter beta probability density function was used to represent the heterogeneity of the persistency among the different nodes in the network. This led to the following equation for $CV_D$ (Methods):

4 $$\begin{aligned} CV_D = CV_L = \sqrt{\frac{(1-{\bar{P}})^2}{{\bar{P}}(2-{\bar{P}})}} \, . \end{aligned}$$

Differently from Eqs. (3), (4) accounts for the statistical distribution of flow intermittency within the river network. The resulting expression for $CV_D$, however, only depends on the mean network persistency because the term $\widetilde{\Delta P}$ of Eq. (2) was in turn expressed as a function of ${\bar{P}}$. The existence of a general link between $\widetilde{\Delta P}$ and ${\bar{P}}$ should not be surprising, since in perennial (or persistently dry) rivers all the nodes necessarily exhibit very similar persistencies—thereby implying that $\widetilde{\Delta P} \simeq 0$ for very high or very low values of ${\bar{P}}$.

**Figure 2:**
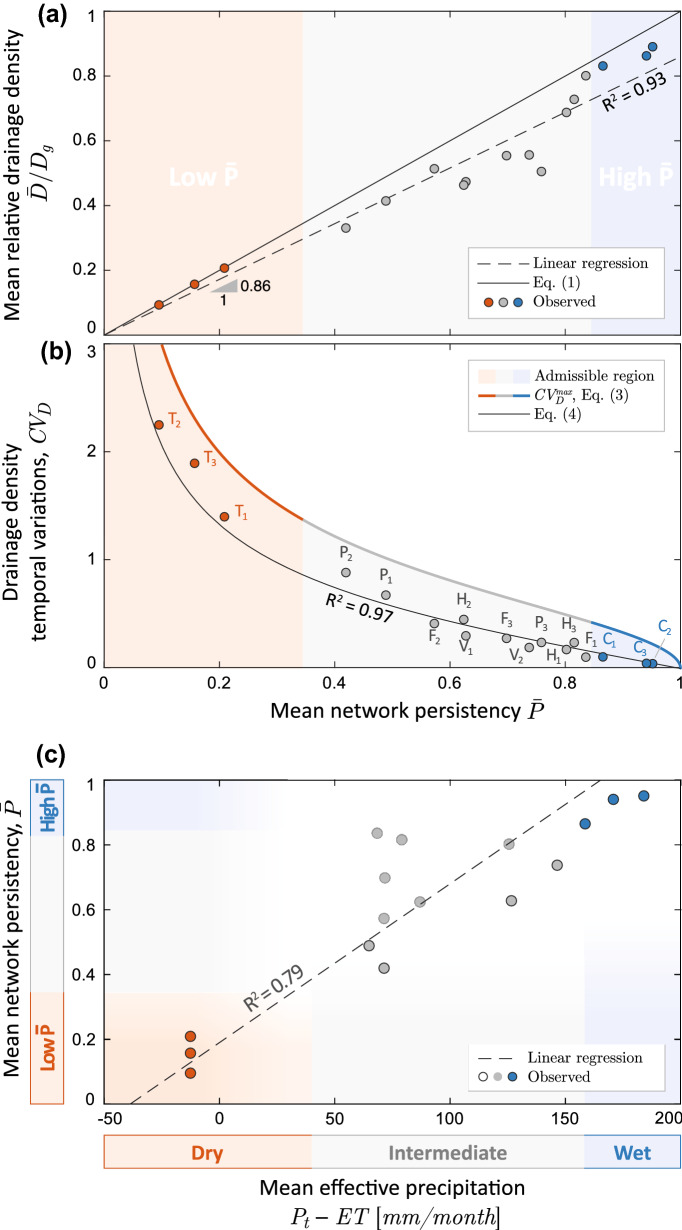
Climatic controls on the active drainage density. (**a**) mean drainage density scaled to the geomorphic drainage density as a function of the mean network persistency, ${\bar{P}}$; (**b**) coefficient of variation of the drainage density as a function of the mean network persistency ${\bar{P}}$. Also shown here are the maximum theoretical limit for $CV_D$ (Eq. (3)) and the value of $CV_D$ predicted by the hierarchical beta-model (Eq. (4)); (**c**) the correlation between mean network persistency and effective precipitation (total precipitation minus potential evapotranspiration ET) during the period when the active network was surveyed (average monthly value among all the months during which at least one field survey was performed). Points with a lighter gray border represent cases in which the surveys are not evenly distributed within the study period (Hubbard Brook and Fernow) as the median time between all the possible pairs of survey dates, $\tau _m$, was smaller than 15 days. $P_3$ was not included in this plot because of the lack of reliable precipitation data.

The theoretical relationships between the statistics of the dynamic drainage density and the mean network persistency were tested using active length data derived from field surveys in our case studies (Fig. 2). As expected, the mean network persistency ${\bar{P}}$ well approximates the temporal average of the active drainage density normalized by the geomorphic drainage density, as implied by Eq. (1) (Fig. 2a). The 1:1 relationship between the spatial average of the mean percentage of time during which a node is active and the temporal average of the normalized mean active length is implied by the linearity of the average operation. Because a 1:1 relationship between ${\bar{P}}$ and ${\bar{D}}/D_g$ is obtained regardless of the specific activation order that underlies the network dynamics, Fig. 2a should not be interpreted as a validation of the hierarchical scheme. The scattering of the points is only due to the fact that the geomorphic drainage density was calculated independently from the number of network surveys based on geomorphic signatures of the landscape (SI). Available data also confirm that the magnitude of $CV_D$ is modulated by the mean network persistency across the entire set of study catchments, with higher values of $CV_D$ and an enhanced sensitivity to ${\bar{P}}$ for low network persistencies (Fig. 2b). Remarkably, the analytical model given by Eq. (4) captures the observed network length variability across the full set of study catchments ($R^2 = 0.97$), proving that the temporal variation of active streams are in fact linked to the mean persistency of the river network. All the experimental points obey to the inequality $CV_D \, < \, CV^{max}_D$, indicating that the observed temporal dynamics of active stream length are properly constrained by the theoretical limit given by Eq. (3). This finite universal limit of the active length standard deviation derives from the contrasting patterns of $CV^{max}_D({\bar{P}})$ and ${\bar{D}}({\bar{P}})$ shown in Fig. 2 (Supplementary Information). The relatively small distance of the experimental points from the theoretical limit given by Eq. (3) suggests that the total active stream length is about as dynamic as it can possibly be (i.e., the *CV* of network length or drainage density is not far from its maximum possible value). Active stream dynamics and temporarily-dry channels are known to modify the distribution of catchment travel times owing to changes in the length of channel and hillslope flowpaths^27,34^, thereby affecting important biogeochemical and ecological properties of rivers across a broad range of hydroclimatic settings^22,35–37^. Thus, temporal variations of the active channel length should be properly taken into account in regional assessments of biogeochemical function of river networks, such as $CO_2$ outgassing and nutrient spiraling^38–42^.

Other relevant implications of our results stem from the identification of the major physical determinants of the mean network persistency. Surface flow is the byproduct of the excess water drained by the upstream contributing catchment, as proposed by^12,21^. Thus, in line with these previous studies, we hypothesize that the mean network persistency represents an important climatic signature of river basins. Accordingly, ${\bar{P}}$, which was poorly correlated with drainage basin areas ($R^2 < 0.01$), was found to be strongly linked to the underlying climatic water balance, here represented by the effective precipitation, $P_t-ET$ (i.e., total precipitation minus the potential evapotranspiration during the surveyed periods), as shown by Fig. 2c. Despite the high correlation between effective precipitation and persistency (overall $R^2 = 0.79$), some scattering appears in the observational data. This scatter is attributed to two factors: (1) relevant inter-catchment differences of geological or morphometric features, as suggested by^21,43^; and (2) an uneven distribution of the survey dates that might bias the relationship between the observed mean network persistency and the average climatic conditions during the study period for some catchments (light grey dots in Fig. 2c). Nevertheless, we found a clear climatic signature with drainage density statistics: lower mean network persistencies and enhanced relative active length variability were observed in the drier catchments (with $CV_D > 1.5$ for $P_t-ET < 40 \,$mm/month, as in the Turbolo site). Conversely, in the wettest site (Coweeta, where $P_t-ET > 160\,$mm/month), the mean network persistency approached unity and network dynamics are smoothed, with $CV_D \rightarrow 0$. This result offers novel insight on the impact of climate on the active stream length variability^44^, and nicely complements existing modeling and empirical approaches for the characterization of the spatial patterns of flow persistency across a landscape^21,31,45^ . Therefore, we believe that the proposed framework can provide useful insight into the impact of climate on the temporal changes of the active drainage density.

**Figure 3:**
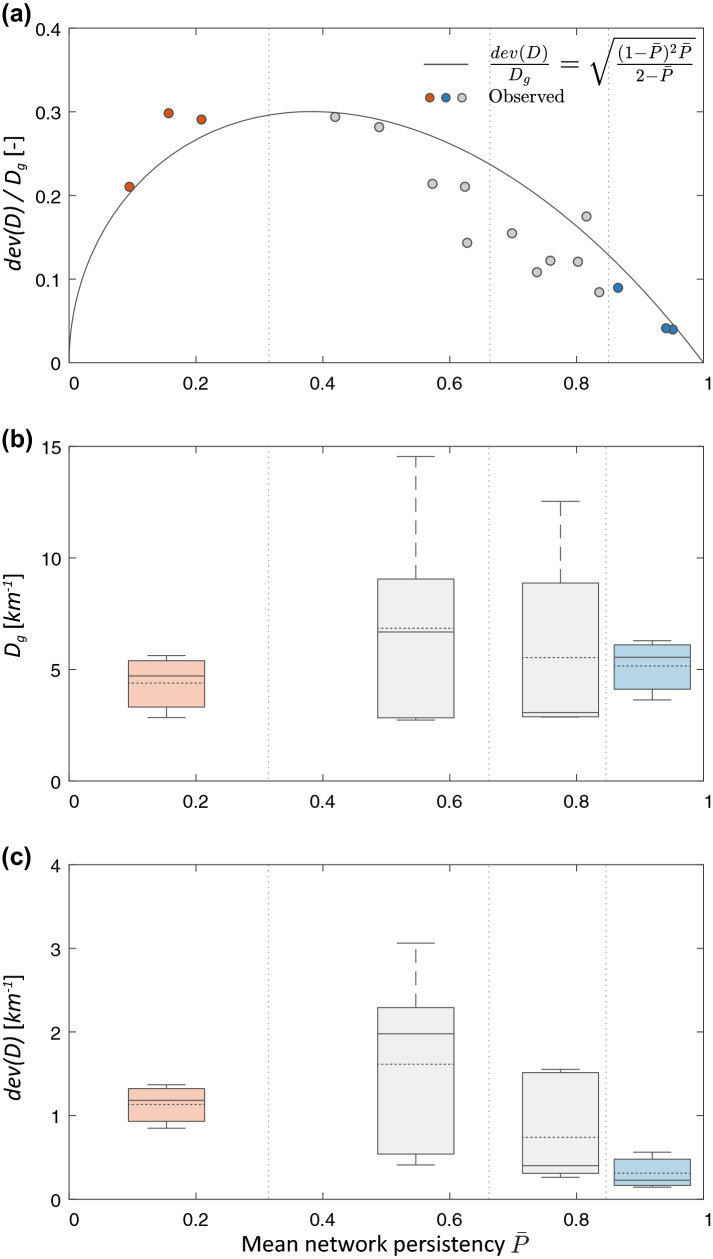
Dependence of key drainage density statistics on the mean network persistency, ${\bar{P}}$; the following statistics are shown in the three panels: (**a**) the standard deviation of the dynamic drainage density, *dev*(*D*), scaled to the geomorphic drainage density, $D_g$; (**b**) the geomorphic drainage density; (**c**) the standard deviation of the active drainage density, that is proportional to the standard deviation of the active length through the contributing area. In panel (**a**) we show a comparison between observed values of $Dev(D)/D_g$ (dots) and the corresponding theoretical prediction of the analytical model as derived from Eqs. (1) and (4) for all the case studies where active lengths were monitored; in panels (**b**,**c**) the box-plots represent the first, second and third quartiles of observed data (solid lines) aggregated for different persistency ranges (as indicated by the dashed lines). The average is shown as a dotted line, while the full range is represented by whiskers.

## Implications for large-scale studies

Several studies have investigated the impact of rainfall regimes on the geomorphic drainage density, $D_g$^46–49^. However, the influence of hydroclimatic drivers on the active fraction of the network is not fully understood. Notwithstanding the variability of $D_g$ across the study catchments (attributed to their geological diversity), our results confirm that the extent of active channel length is linked to the amount of excess precipitation in the contributing catchment, as previously proposed in the literature^12,31^. This suggests the emergence of distinctive ecohydrological patterns within different climatic regions of the world. The largest temporal variations in the active stream length were found in the sites characterized by intermediate climates (i.e., $40< P_t-ET < 100\,$mm/month and $0.4< {\bar{P}} < 0.6$), where the standard deviation of the active length and the geomorphic drainage density are concurrently higher (Fig. 3). Crucially, for intermediate values of $P_t-ET$ both the overall dynamic channel length and its internal heterogeneity are enhanced. In these settings, in fact, the observed local persistencies ranged from 0 to 1 (Supplementary Information) revealing the presence of multiple expansion/contraction cycles that operate with diverse frequencies, from individual storms to seasons. This behaviour generates a gradient of spatially heterogeneous habitats, from predominantly terrestrial to lentic, widening the spectrum of adaptation strategies and increasing biodiversity^22^. In the driest site (Turbolo), the relative variability of *D* is much higher than in all other settings (Fig. 2b), as almost the entire network is dynamic. However, the mean active length is typically reduced because of the predominance of dry reaches experiencing episodic activation. The lower persistencies induced by limited water availability entail shorter dynamical lengths of streams, regardless of the pronounced temporal changes of flow conditions experienced by dry rivers^50^. The ability of our analytical framework to quantitatively describe this wide spectrum of behaviours suggests the general applicability of the proposed approach within large-scale studies and makes it a sound basis for developing more specific numerical tools aimed at interpreting or predicting channel network dynamics.

Our analysis elucidates the major implications of the observed hierarchical behaviour of temporary streams on the spatial statistics of the node states and the temporal dynamics of the flowing length. In particular, temporal changes of the active drainage density are linked to the catchment water balance through the mean persistency of the whole network, whereas observed spatial patterns of flowing streams maximize the spatial correlation of the node states (wet vs. dry). The internal heterogeneity of the node persistency, though reflecting landscape complexity, is also modulated by climate and proves to be particularly enhanced under intermediate climatic conditions, leading to larger dynamical channel lengths in these settings. Furthermore, our study indicates that, because changes in wetness are generally synchronized, stream networks tend to be highly dynamic (i.e., they are nearly as dynamic as they can possibly be). Consequently, temporal variations of the active stream length and their climatic controls should be properly taken into account for accurate regional or global assessment of biogeochemical and ecological function of stream networks, particularly with changing climate.

## Methods

## Statistical moments of active length and drainage density

A river network is here represented as a sequence of *N* connected nodes, where the status of the node *i* ($i \in (1,N)$) identifies the hydrologic conditions within a stretch of stream length $\Delta l_i$. Without loss of generality, here the reach associated to the node *i* is not the link with one of the adjacent nodes but rather a stretch that embeds the node *i*. Accordingly, its length $\Delta l_i$ is given by the sum of the semi-distances between the node *i* and all the connected neighbouring nodes. The status of each node corresponds to the status of the associated stretch and is represented by a binary random variable $X_i$, with $X_i=1$ if the node experiences flowing water and $X_i=0$ otherwise. Thus, the active network length can be expressed as: $L = \sum _{i=1}^N \Delta l_i \; X_i$. If, without loss of generality, we assume $\Delta l_i = \Delta \,l=const$ (i.e., equally spaced nodes), the mean active length can be calculated as:

5 $$\begin{aligned} {\bar{L}} = E[L] = N \; \Delta l \; E[X] = L_{max} \; {\bar{P}} \, , \end{aligned}$$

where $E[-]$ denotes expectation, ${\bar{P}}$ is the mean network persistence and $L_{max} = N \Delta l$ is the geomorphic length (i.e. the maximum length of the network). Equation (5) and the Hortonian definition of drainage density, $D=L/A$ ($A=$ catchment area) are then used to derive Eq. (1) in the main text (see Supplementary Information).

In this framework, the variance of the active length, *Var*(*L*), is linked to the covariances $Cov(X_i,X_j)$ between the status of all the pairs of nodes as:

6 $$\begin{aligned} Var(L) = \Delta l^2 \sum _{i=1}^N \sum _{j=1}^N Cov(X_i, X_j) \, . \end{aligned}$$

Under the assumption of hierarchical activation of the nodes^51^, the covariance between $X_i$ and $X_j$ only depends on the persistency of the nodes *i* and *j*:

7 $$\begin{aligned} Cov(X_i, X_j) = Cov(X_j, X_i) = \left\{ \begin{matrix} P_j ( 1 - P_i ) \; if \; P_i \ge P_j\\ P_i ( 1 - P_j ) \; otherwise \end{matrix}\right. \end{aligned}$$

Without loss of generality, we enumerate the nodes in a way such that $P_i \ge P_j$ whenever $i < j$. Combining Eqs. (6) and (7), *Var*(*L*) can be thus expressed as (Supplementary Information):

8 $$\begin{aligned} \begin{aligned} Var(L)&= \Delta l^2 \left[ \sum _{i=1}^N P_i (1-P_i) + 2 \sum _{i=2}^N \sum _{j=1}^{i-1} P_j(1-P_i) \right] \\&\simeq L_{max}^2 \left[ {\bar{P}} (1-{\bar{P}}) + \widetilde{\Delta P} \right] \, , \end{aligned} \end{aligned}$$

which leads to Eq. (2) in the main text. If the local persistency is uniform ($P_i = {\bar{P}}$), then $\widetilde{\Delta P} = 0$ in Eq. (8); under these circumstances, combining Eqs. (8) and (5) we obtain the upper theoretical limit for the coefficient of variation of the drainage density (and of the active length) expressed by Eq. (3). This limit also holds for non-hierarchical networks, since any departure from the hierarchical behavior would further decrease the observed $CV_L$ and $CV_D$ (see below).

Empirical data suggest that the local persistency along a network can be in most cases represented by a beta distribution with the first shape parameter equal to 1. Under this assumption, the term $\widetilde{\Delta P}$ in Eq. (8) reads (Supplementary Information):

9 $$\begin{aligned} \widetilde{\Delta P} = - \frac{{\bar{P}}(1-{\bar{P}})}{2-{\bar{P}}} \end{aligned}$$

and combining Eqs. (9) and (2), $CV_D$ takes the form shown in Eq. (4) of the main text.

### The hierarchical activation scheme maximizes the spatial correlations and the temporal variability of *L*

In the general case, the covariance between $X_i$ and $X_j$ can be expressed as (Supplementary Information):

10 $$\begin{aligned} Cov(X_i, X_j) = P_j (1-P_i) - P_{01} \, , \end{aligned}$$

where $P_i$, $P_j$ are, respectively, the marginal probabilities that the nodes *i* and *j* are active (i.e. the persistency of the nodes *i* and *j*) and $P_{01} = Prob[X_i = 0 \; and \; X_j = 1]$ is the joint probability that, at a given time, the node *i* is dry and the node *j* is active. As the covariance is symmetrical, without loss of generality let us assume $P_i \ge P_j$. For hierarchical networks, the less persistent node (*j*) can be active only if the more persistent node (*i*) is active. This implies $P_{01} = 0$ in Eq. (10), thereby leading to the maximization of $Cov(X_i, X_j)$ $\forall \, (i,j)$. Thus, for a given set of local persistencies, the hierarchical behaviour maximizes the covariance and the spatial correlation of the states of each pair of nodes. This implies that the mean spatial correlation within the entire network is maximized. Consequently, when the node activation in a temporary stream is hierarchical, the variance of the active length (and that of the drainage density) is maximized, as per Eq. (6).

### Study catchments and empirical data

Our empirical dataset refers to 19 different catchments in Europe and USA, spanning a wide range of latitudes, climates, land covers, geologies and sizes (see Fig. 1 and Supplementary Information). This selection includes most of the monitoring sites where observational data on the temporal changes of the active network length are available for more than one season with an average resolution exceeding one survey per month. In the USA, 12 catchments (indicated with the capital letters *P*, *H*, *F* and *C* in Fig. 2 and in the main text) were selected that cover 4 different physiographic provinces of the Appalachian Highlands with different bedrock types and structure. The mean annual precipitation is between 1000 and 1800 mm, with catchment areas ranging between 12 and 70 ha. Maps of the flowing network were obtained from visual inspection during 7 surveys in 2015-2016^28^. In northern Italy, the Valfredda catchment (labelled as *V*) has been monitored 9 times during the summer and fall of 2018. This catchment has an area of 5.3 $\mathrm{km}^2$ and highly variable geology consisting of morains, tallus slopes, and carbonate and siliciclastic bedrock. The climate is alpine, with a mean annual precipitation of approximately 1500 mm^27^. In spite of the wet climate, the stream network contains three reaches that are permanently disconnected to the outlet. This is the result of a strong physiographic heterogeneity of the contributing catchment, which determine a local increase of the soil storage capacity in the northern portion of the basin. Three additional catchments in southern Italy (labelled as *T*) were also considered, with drainage areas of 67, 48 and 24 ha. Therein, the active network has been monitored on average 38 times in 2019 and early 2020. Despite significant inter-catchment morphological heterogeneities, these sites are all characterized by sandy-conglomerate formations and a fractured crystalline-metamorphic bedrock. The climate of the region is Mediterranean, with a mean annual precipitation around 1300 mm and hot, dry summers. The largest study catchments of this paper are the Attert (247 $\mathrm{km}^2$, Luxembourg) and the Seugne (920 $\mathrm{km}^2$, western France), where available data quantify flow presence in a number of sparse nodes (182 for the Attert and 40 for the Seugne). The Attert catchment, consisting of slate, marls, and sandstones, was monitored using water presence sensors at sub-hourly temporal resolutions from 2013 to 2017. In this region, the climate is characterized by mild summers and wet winters^52^ and the mean annual precipitation is 850 mm. The Seugne catchment was monitored monthly via visual inspections from 2012 to 2020. The main geologic units of this catchment include limestones, carbonates sedimentary rocks and sand, whereas the climate is Marine West Coast, with a mean annual precipitation around 850 mm evenly distributed throughout the year. The data of the Attert and the Seugne were used only for the validation of the hierarchical model because the active lengths were not monitored.

### Calculation of relevant hydrologic and climatic indexes

For each case study where active stream maps were available, the network was represented by a large number of equally spaced nodes (on average 1250 nodes per $\mathrm{km}^2$ of contributing area), that were assumed as representative of the hydrological status of a homogeneous reach in between two or more connected nodes. The nodes were designed to represent the full geometry of the geomorphic network, here defined as the maximum possible extension of the stream network. While there are small differences across the study sites in the procedure used to estimate the geomorphic network, in all cases the latter was obtained from field surveys and included the sites that were dry during the surveys but showed clear evidence of channelization. For each field survey, the active length was calculated as the sum of the individual lengths associated with all the active nodes in the network. Then, for each catchment, the sample mean and variance of *L* were used to estimate ${\bar{L}}$ and *var*(*L*). The local persistency of node *i*, $P_i$ was calculated as the fraction of times in which that node was observed as active during the surveys. Likewise, the mean network persistency ${\bar{P}}$ was then computed as the spatial average of the local persistency $P_i$. For each catchment, the time between any possible pair of mapping dates was also calculated, and the median time $\tau _m$ was estimated to assess the potential bias introduced by an uneven distribution of the dates when active lengths were mapped. The climatic water balance was analyzed for each case study, and the effective precipitation, $P-ET$, was calculated as the difference between precipitation and potential evapotranspiration (Supplementary Information).

### Accuracy of the hierarchical model 

The hierarchical model assumes that the activation of the nodes follows a descending persistency order. This implies that, at any given time, there exists a persistency threshold $P^*$ that allows a separation between the dry nodes ($P<P^*$) and the wet nodes ($P \ge P^*$). Accordingly, hierarchical active networks were constructed assuming that, at any given time *t*, each node was active if and only if its local persistency was bigger than a suitable, time-dependent threshold $P^*(t)$. For each field survey, $P^*$ was calculated as the exceedance probability of the corresponding observed number of active nodes. To compare observed and hierarchical active networks, a confusion matrix was computed for each node of each study catchment. The modeled and observed states of each node within all the field surveys were then compared. The spatially and temporally averaged accuracy of the hierarchical model was then calculated as

11 $$\begin{aligned} Accuracy = \frac{TP + TN}{TP + TN + FP + FN} \end{aligned}$$

where TP, TN, FP and FN indicate, respectively, the true positives (i.e. the number of nodes and times for which the status of a node is correctly modeled as active), the true negatives, the false positives and the false negatives. An accuracy of $100\%$ means that the hierarchical model was able to correctly model the status of all the nodes in the network during all the field surveys. The local accuracy shown in the insets of Fig. 1b was defined analogously, the only difference being that it refers to a single node, instead of the whole network.

## Supplementary Information

Supplementary Information.

## Data Availability

The network monitoring data analyzed in this study is publicly available at^53^. All the data about the Attert catchment can be found in^54^. Data about the Seugne catchment is part of the ONDE dataset^55^. Additional information about the catchments can also be found in the Supplementary Information.
