# Supplementary material for: Hierarchical climate-driven dynamics of the active channel length in temporary streams
Source: Sci Rep. 2021 Nov 2;11:21503. doi: 10.1038/s41598-021-00922-2 (PMC8563734; doi:10.1038/s41598-021-00922-2)
Supplement: Supplementary file 1 — Supplementary Information. [file 41598_2021_922_MOESM1_ESM.pdf]

Hierarchical climate-driven dynamics of the active channel length in temporary streams - Supplementary information

Gianluca Botter, Filippo Vingiani, Alfonso Senatore,
Carrie Jensen, Markus Weiler, Kevin McGuire,
Giuseppe Mendicino, Nicola Durighetto

June 2021

1 Supplementary Methods, Figures and Tables

1.1 Study catchments

The empirical dataset used in the paper comprises different experimental sites in Europe and USA. A summary description of each study site is reported below. Tables S1 reports key information about the field surveys performed in each study catchment. The long term climatic, hydrological and geo-lithological characteristics of the sites are reported in tables S2 and S3. Table S4 contains all the data relative to the study periods, as shown in Figures 2 and 3 of the main text..

Valfredda - The Valfredda catchment is located in the southern side of the Alps, in northern Italy. It has a contributing area of 5.3 km^2 and its altitudes range between 1500 and 3000 $ma.s.l.$, with steep slopes. The geomorphic network is 16.8 km long, corresponding to a geomorphic drainage density D_g of 3.16 km^{-1}. The geology and the physiography of the catchment are quite heterogeneous as reflected by the spatial variability of the local persistency throughout the network. Deposits of gravel and rocky debris dominate the upper part of the catchment, generating temporary channels with low persistency. In the lower part of the catchment, instead, the bedrock is covered by a vegetated soil layer. therein, the drainage density is higher and the streams are more persistent and dynamic. Conversely, in the central-eastern part of the catchment, the dolomite bedrock is exposed, generating dynamical streams with high slopes and a low persistency. The catchment experiences a typical alpine climate, with a mean annual precipitation of approximately 1500 mm. The summers are short and wet, with high rainfall frequencies, while the winters are long, cold and dominated by snow input. The active portion of the river network has been surveyed 14 times from July 2018 to May 2019; from Dec 2018 to may 2019, the network remained almost steady due to the winter freezing. The analysis of the available

data has been performed during the whole survey period (V_1) and then focussing only on the summer and fall seasons, that represent the period during which the network is more dynamic (July-Nov 2018, V_2). For more detailed information about the Valfredda catchment the reader is referred to [28].

Turbolo - The Turbolo creek is a small river located in southern Italy, where the climate is Mediterranean with hot summers; its drainage area is approximately 7 km^2 at the outlet of Fitterizzi, with elevations ranging between 183 and 1005 $ma.s.l.$. The empirical data used in this study refers to two sub-basins in the northern part of the catchment, with contributing areas of 0.67 and 0.48 km^2 (catchments T_1 and T_3, respectively). A third catchment (T_2), nested into T_1, has also been identified to capture the effects of the internal heterogeneity of geology and land cover in the study area. The T_2 catchment drains an area of 0.23 km^2 only. The Mediterranean climate of the area consists in hot and dry summers with wet, mild winters. The annual precipitation is approximately 1200 mm, but due to high evapotranspiration rates a complete dry-down of the network is observed during the summer. The T catchments are dominated by silty marly clays with low permeability, that generate (jointly with the aridity of climate) streams with very low persistency. The geomorphic drainage densities in this site range between 2.8 and 5.6 km^2. The upper part of T_1 is composed by sandy-conglomerate formations, in which the higher permeability produce a relative increase in the persistency of the stream network. The catchments T_1 and T_2 have been monitored for 35 times from April 2019 to January 2020, while T_3 was monitored for 42 times from May 2019 to January 2020.

Hubbard Brook - Three US catchments considered in this study pertain to the Hubbard Brook Experimental Forest of New Hampshire (New England). In the paper, they have been identified as H_1, H_2 and H_3, and their contributing areas are 0.14, 0.25 and 0.42 km^2, respectively. The mean altitude of these catchments ranges between 630 and 740 $ma.s.l.$, with average slopes of 28%. The stream networks of these catchments are the densest of our dataset, with geomorphic drainage densities of 12.5, 14.5 and 8.9 km^{-1}, respectively. The geology of the sites is mainly composed of shists and granulites, covered by a mantle of sandy loams. The humid continental climate of the area provides a mean annual precipitation of 1400 mm, with cold snowy winters and mild summers. For each catchment, 7 visual monitoring of the active network have been carried out in June and July 2015, spanning a wide range of hydroclimatic conditions. More information on this catchments is available in [27].

Fernow - The Fernow Experimental Forest is located in the Appalachian Plateau of West Virginia (USA). In this site, three study catchments (F_1, F_2, F_3) have been selected, with drainage areas of 0.14, 0.16 and 0.37 km^2, respectively. These catchments have a mean altitude of approximately 800 $ma.s.l.$, with slopes around 30%. Field observations revealed geomorphic drainage densities of 2.9 km^{-1} with local persistencies varying in the full range between 0 and 1. The bedrock is composed of shales and sandstones, with a soil cover consisting mainly of silt loams. The average annual precipitation is approximately 1450 mm, with rainy summers and mite winters. Snowfalls are common but usually

limited and isolated. From May to December 2016 a total of 7 field surveys per catchment were carried out. More information on this study site can be found in [27].

Potts and Poverty - Three study catchments are located in the Valley and Ridge physiographic provinces of Virginia (USA). Two of them (P_1 and P_2) are part of the Poverty Creek, while the third (P_3) belongs to the South Fork Potts Creek, 25 km northern of P_1 and P_2. These catchments have a contributing area of 0.25, 0.35 and 0.73 km^2, respectively. P_1 and P_2 have and average elevation of 750 $ma.s.l.$, a mean slope of 33% and a geomorphic drainage density of 7 km^{-1}. The Potts Creek catchment, instead, has a higher elevation (1030 $ma.s.l.$) and gentler slopes (27%). Its geomorphic drainage density is 2.9 km^{-1}. Bedrocks are mainly composed by standstones in all the catchments, whereas shales and siltstones can be found in the P_1 and P_2 sites. The average annual precipitation in the area is close to 1000 mm, remarkably lower than all the other USA catchments considered in this study (and comparable with the T sites in southern Italy). Unlike the Turbolo Creek, however, the drainage networks do not experience summer dry down due to the lower evapotranspiration rates. The active network in these catchment has been monitored by visual inspection 7 times between September 2015 and March 2016. More information about these study sites can be found in [27].

Coweeta - The C_1, C_2 and C_3 catchments are located in the Coweeta Hydrologic Laboratory, North Carolina (USA). They are the highest and steepest USA sites considered in this study, with mean elevations between 800 and 1100 $ma.s.l.$ and slopes of about 50%. These sites have areas of 0.12, 0.33 and 0.40 km^2, respectively, and drainage densities ranging between 3.6 and 6.2 km^{-1}. The catchments lie on the Southern Blue Ridge Mountains, which mainly consist of exposed metamorphic rock (biotite gneiss and amphibolite). The climate is generally warm and humid, with temperatures above $0°C$ all year round. The precipitation is greatest in winter and early spring, with an average annual precipitation of about 1800 mm. The field campaign for detecting the active stream network in these sites were carried out from November 2015 to December 2016, and consisted in 7 field surveys for each catchment. More information about these study sites can be found in [27].

Attert - The Attert catchment (cathcment code A) covers an area of 247 km^2 between Luxemburg and Belgium. The altitude ranges between 245 and 550 $ma.s.l.$ and the geology is dominated by slate, marls and sandstone, while lithology is driven by land use (mainly forest and agricolture). The climate is characterized by wet winters and mild summers, with an average annual precipitation of 900 mm uniformly distributed troughout the year (*58*). Different types of sensors were employed in this catchment (time-lapse photography, conductivity sensors and water level gauging stations) to monitor flow presence in a set of 182 nodes along the network. These sensors recorded water presence at a 30-minute time interval from 2013 to 2017. The sparse arrangement of the sensors along the network didn't allow a proper calculation of the active length. Therefore this data was only used to validate the hypothesis of hierarchical activation of the stretches. Detailed information about this catchment can be found

in [56].

Seugne - The Seugne river is located in the Charente-Maritime département in west France. The catchment (hereafter coded as S) has a contributing area of 920 km^2, with elevations that span between 8 and 160 $ma.s.l.$. The geology is composed of limestones, carbonates, sedimentary rocks and sand. The climate is Marine West Coast. The active network was monitored in the catchment as part of the Onde campaign. Visual inspection observations were employed determine water presence in a set of 40 predetermined nodes, at a monthly interval from 2012 to 2020. As for the Attert catchment, this data was only used for validating the hierarchical hypothesis because the sparse monitoring points did not allow the calculation of the active length. More information about the field campaign can be found in (*59*).

Table S1: Characteristics of the field campaigns carried out for the monitoring of the active stream network and accuracy of the hierarchical model.

**Catchment code**	Area $[km^2]$	**Survey type**	**# of surveys**	**Survey period**		**# of noeds**	τ_m $[days]$	**Accuracy**	Reference (watershed number[a])
A	247	ER sensors	33770	Aug-2015	Jul-2017	182	—	94%	Kaplan et al. 2019
C_1	0.12	Visual	7	Nov-2015	Dec-2016	323	146	100%	Jensen et al. 2017 (WS 18)
C_2	0.33	Visual	7	Nov-2015	Dec-2015	365	133	100%	Jensen et al. 2017 (WS 34)
C_3	0.4	Visual	7	Dec-2015	Dec-2016	707	128	100%	Jensen et al. 2017 (WS 32)
F_1	0.14	Visual	7	Jun-2016	Dec-2016	175	13	100%	Jensen et al. 2017 (WS 13)
F_2	0.16	Visual	7	May-2016	Dec-2016	243	14	100%	Jensen et al. 2017 (WS 10)
F_3	0.37	Visual	7	May-2016	Dec-2016	489	14	100%	Jensen et al. 2017 (WS 4)
H_1	0.14	Visual	7	Jun-2015	Jul-2015	861	13	100%	Jensen et al. 2017 (WS 6)
H_2	0.2	Visual	7	Jun-2015	Jul-2015	1429	10	100%	Jensen et al. 2017
H_3	0.43	Visual	7	Jun-2015	Jul-2015	2218	14	100%	Jensen et al. 2017 (WS 3)
P_1	0.25	Visual	7	Sep-2015	Jan-2016	1045	41	100%	Jensen et al. 2017
P_2	0.35	Visual	7	Sep-2015	Dec-2015	1225	23	100%	Jensen et al. 2017
P_3	0.73	Visual	7	Aug-2015	Mar-2016	906	61	100%	Jensen et al. 2017
S	920	Visual	78	Apr-2012	Oct-2019	40	—	96%	ONDE Dataset
T_1	0.63	Visual	35	Apr-2019	Jan-2020	368	75	99%	This study
T_2	0.23	Visual	35	Apr-2019	Jan-2020	173	75	99%	This study
T_3	0.46	Visual	42	May-2019	Jan-2020	205	67	99%	This study
V_1	5.27	Visual	14	Jul-2018	May-2019	504	82	97%	This study
V_2	5.27	Visual	9	Jul-2018	Nov-2018	504	40	96%	Durighetto et al. 2020

[a] If applicable, for gauged catchments at the experimental forests with a designated watershed number

Table S2: Summary of the main climatic and hydrological characteristics of the study catchments (NA = data not available). Note that the mean climatic attributes reported here are long term averages and do not refer to the study period only.

**Catchment code**	**Area** $[ha]$	**Mean elevation** $[ma.s.l.]$	**Mean temperature** $[^{\circ}C]$	**Mean precipitation** $[mm/month]$	**Mean evapotranspiration** $[mm/month]$	**Mean streamflow** $[mm/month]$	**Runoff coefficient**	**Aridity index**
A	24700	350	9.6	74.6	51.3	36.3	0.47	1.45
C_1	12	823	13.7	226.0	68.2	172.5	0.76	3.31
C_2	33	1019	13.8	229.7	77.8	160.5	0.70	2.95
C_3	40	1052	11.9	285.3	56.1	214.4	0.75	5.08
F_1	14	773	15.6	144.1	83.0	58.1	0.40	1.74
F_2	16	767	15.6	144.9	86.4	40.4	0.28	1.68
F_3	37	822	15.6	146.5	86.4	39.5	0.27	1.69
H_1	14	690	16.2	163.7	104.2	87.2	0.53	1.57
H_2	20	740	17.1	209.8	119.0	117.1	0.56	1.76
H_3	43	632	16.2	162.8	103.4	85.2	0.52	1.57
P_1	25	750	10.1	73.2	55.8	69.4	0.95	1.31
P_2	35	729	14.2	77.4	69.0	32.1	0.41	1.12
P_3	73	1029	NA	NA	36.4	57.6	NA	NA
S	91950	100	17.3	62.7	NA	NA	NA	NA
T_1	63	263	17.2	87.8	109.5	47.6	0.54	0.80
T_2	23	250	17.2	87.8	109.5	47.6	0.54	0.80
T_3	46	358	17.6	88.3	112.9	48.9	0.55	0.78
V_1	527	2250	7.6	152.7	47.8	86.4	0.57	3.19
V_2	527	2250	13.8	197.8	64.6	119.0	0.60	3.06

Table S3: Summary of the geology and soil use of the study catchments.

**Catchment code**	**Mean elevation** $[m a.s.l.]$	**Geology**	**Soil use**
A	350	Marl, slate and sandstone	Annual crops, fruit trees and mixed forests
C_1	823	Gneiss	Deciduous forest
C_2	1019	Gneiss	Deciduous forest
C_3	1052	Gneiss	Deciduous forest
F_1	773	Shale rocks	Deciduous forest
F_2	767	Shale rocks	Deciduous forest
F_3	822	Shale rocks	Deciduous forest
H_1	690	Mudstone and shists	Deciduous forest
H_2	740	Mudstone and granite	Evergreen and mixed forest
H_3	632	Shists and mudstone	Evergreen and deciduous forest
P_1	750	Shale rocks	Deciduous forest
P_2	729	Shale rocks	Deciduous forest
P_3	1029	Sandstones	Deciduous forest
S	100	Limestones	Annual crops, fruit trees and deciduous forests
T_1	263	Clays	Annual crops and fruit trees
T_2	250	Clays	Annual crops and fruit trees
T_3	358	Clays	Annual crops and steady meadows
V_1	2250	Limestone and sandstone	Natural pastures, coniferous forest and rocks
V_2	2250	Limestone and sandstone	Natural pastures, coniferous forest and rocks

1.2 Local persistency of the study catchments

Figure S1 shows the frequency distribution of the local persistency of the nodes within each study catchment (histograms). The plots also show as a solid line the fitted Beta distribution (see equation (S11)). Furthermore, the corresponding spatial maps of local persistency are represented in Figure S2.

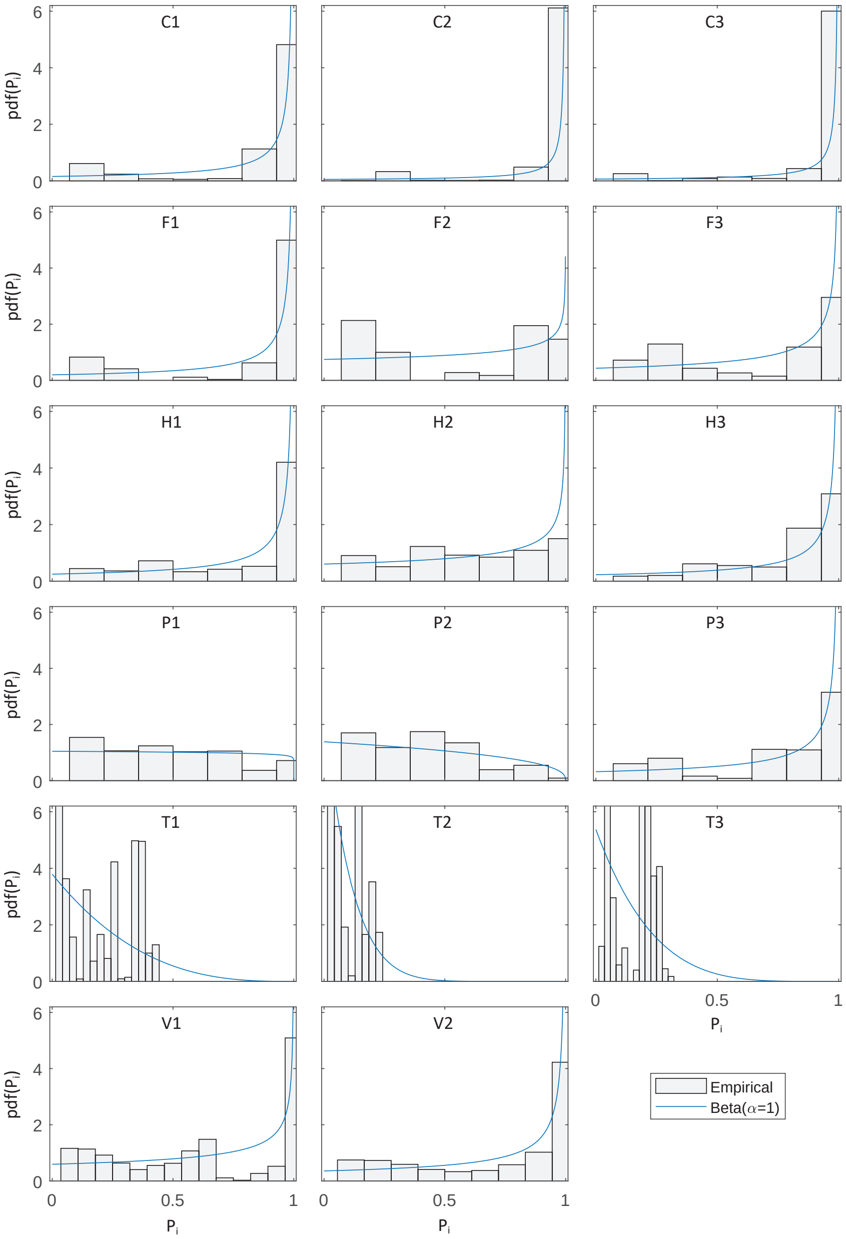

Figure S1: Probability Density Function of the local persistency P_i of the study catchments.

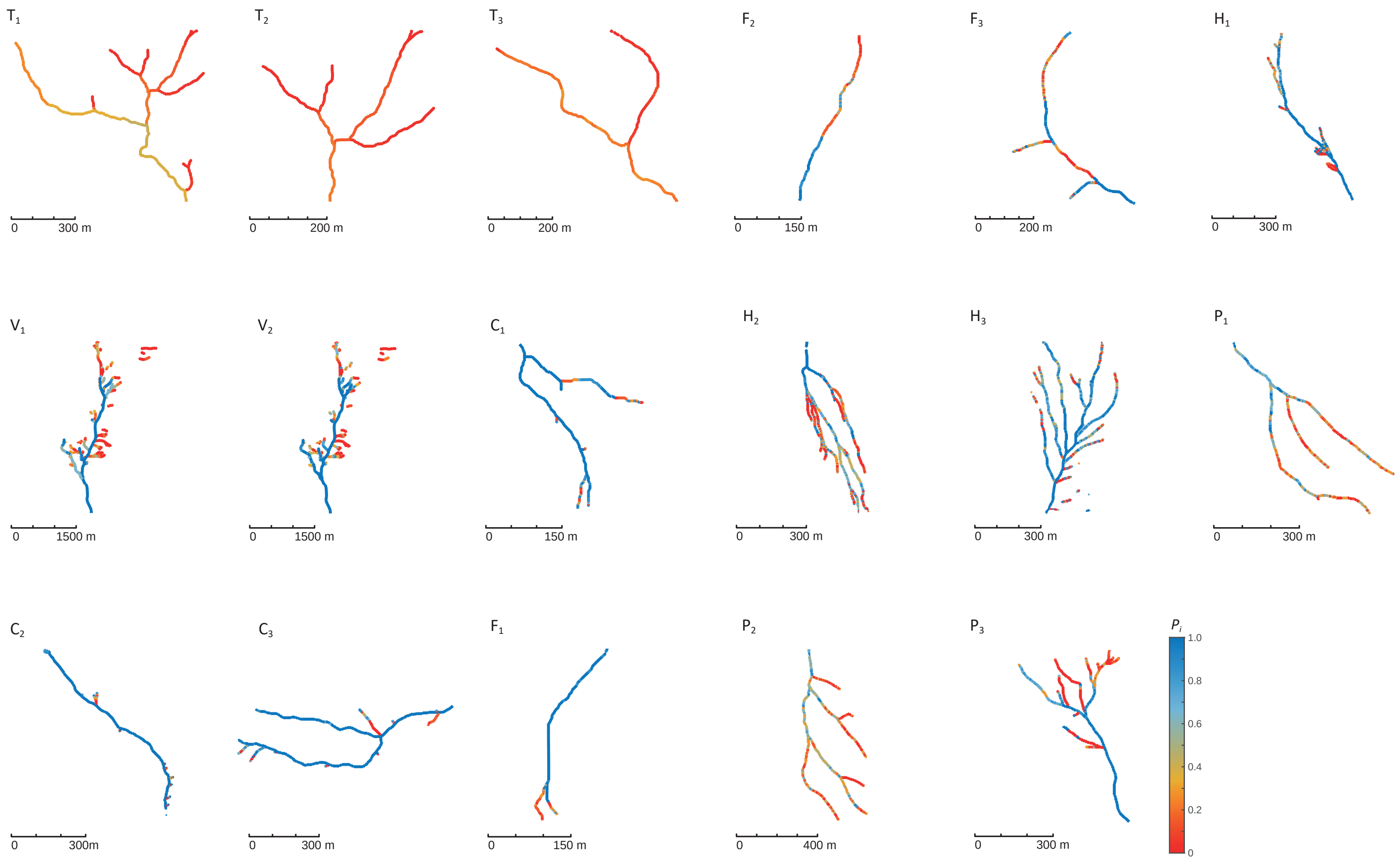

Figure S2: Maps of local persistency of the study catchments.

1.3 Climatic and morphologic features of the study catchments

Figure S3 summarizes the main climatic and hydrological characteristics of the study catchments reported in Table S2. The figures indicate that the study catchments cover a wide range of climatic conditions in terms of mean precipitation, evapotranspiration, aridity, temperature, catchment area and elevation. Therefore, the dataset can be considered as representative of a broad range of climatic and morphologic settings.

1.4 Methods underlying Figure 2c in the main text

Calculation of $P_t - ET$

The effective precipitation $P_t - ET$ is used in this paper to quantify the specific (per unit catchment area) catchment water availability. $P_t - ET$ is calculated as the difference between the total precipitation and the potential evapotranspiration temporally averaged over a suitable reference period. Specifically, the reference period consists of the months during which the field surveys for the network monitoring have been carried out. If a field survey was performed in the first 8 days of the month, the month before the first survey of the campaign has been included in the computation of $P_t - ET$. For the Italian catchments, climatic data necessary for the calculation of $P_t - ET$ were retrieved from the nearest Regional Environmental Protection Agency weather station (the Falcade station of ARPAV for the V catchment and the Fitterizzi station of ARPACAL fot the T catchment). Precipitation and streamflow data in the H, F and C catchments, instead, were provided by the Hubbard Brook Experimental Forest (*60*), the Fernow Experimental Forest (*61*) and the Coweeta Hydrologic Laboratory (*62*) respectively. Data from the NOAA meterologic station US1VAMN0025 were also used for the P_1 and P_2 catchments. No precipitation data were available for P_3 instead. Monthly potential evapotranspiration data for all the US catchments were provided by USGS (*63*). This data were estimated at a 1 km spatial scale using the SSEBop model, starting from climatic and morphological data (*64*). Instead, the Penman-Monteith equation was used to estimate the potential evapotranspiration in the T and V catchments, based on available climatic data (*65*).

Calculation of τ_m

The interarrival between any pair of field surveys is here defined as the time elapsed between the dates of the corresponding surveys. For each study catchment, the interarrival between each couple of survey dates has been calculated (including all the couples of non-consecutive surveys). Then, the median interarrival, τ_m, has been computed to quantify to what extent a field campaign was biased by the presence of several surveys concentrated in a relatively short sub-period of the whole survey period (shown in Table S1). The median interarrival was chosen instead of the average interarrival to reduce the effect of field survey that were carried out many months later than all the other surveys. It was found that if τ_m is too small, the surveys reflect the hydrological conditions

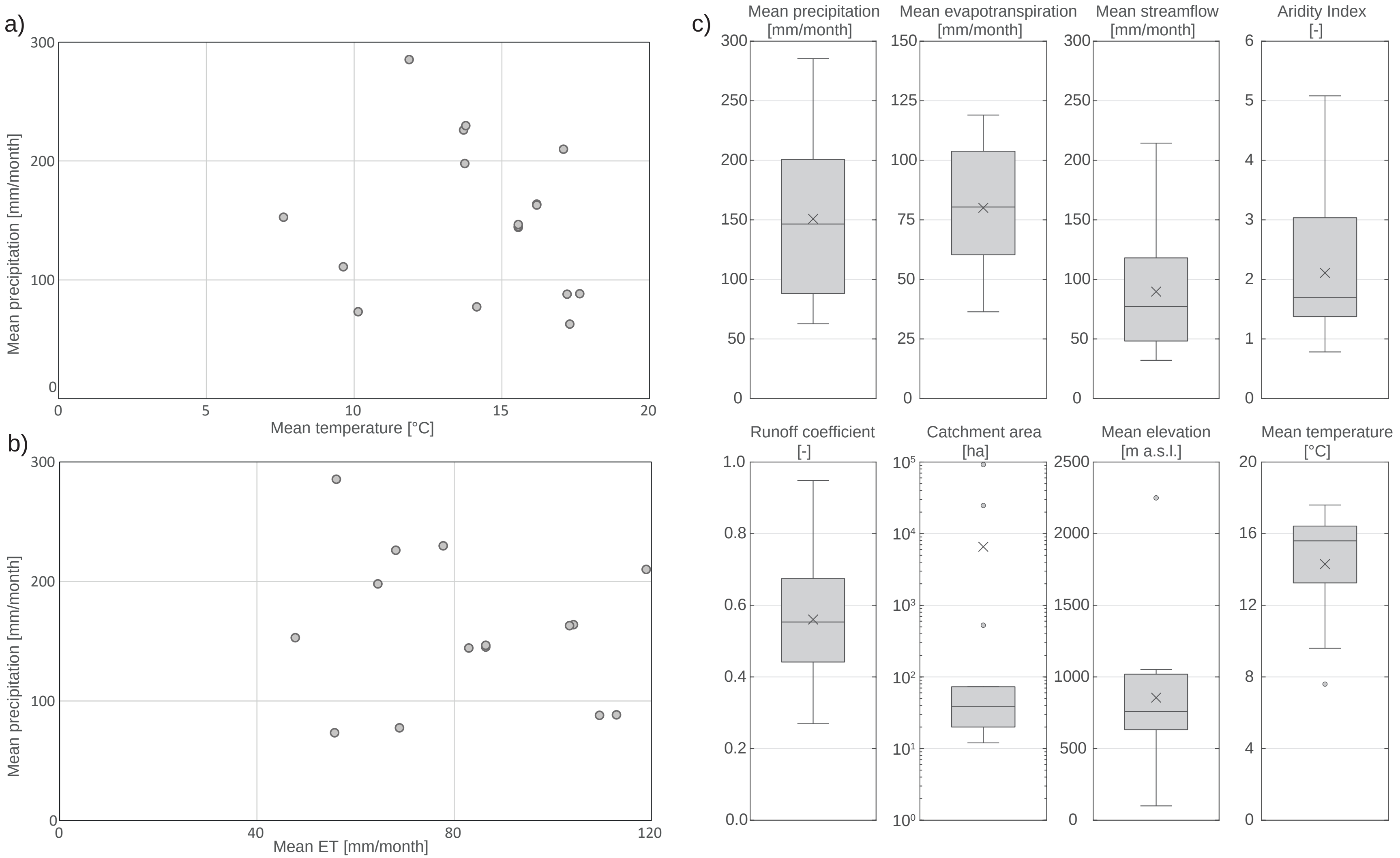

Figure S3: Representation of the study catchments in the Whittaker plot (mean annual temperature vs mean annual precipitation, panel a). Also shown is the scatter plot of the mean evapotranspiration and the mean annual precipitation across all the study sites (panel b). Panel c) shows box plots of the main climatic and morphologic characteristics of the study catchments.

observed during the months in which most of the surveys were performed and not the average climatic conditions over the whole study period. Considering that the mean frequency of the surveys is of the order of one survey per month, in this study it was assumed that τ_m must be greater than > 15 *days* to guarantee that the active lengths observed in correspondence of the field surveys can be properly linked to the average climatic conditions observed within all the months during which at least one survey was performed. The calculated values of τ_m for all the study catchments are reported in Table S1.

1.5 Performance of the hierarchical model

The accuracy of the hierarchical model on each of the study networks is calculated by means of equation (11) of the methods. Figure S5 shows the maps of the local accuracy of the hierarchical model for each study catchment. The Figures indicate that the local accuracy is close to 100% in most cases. The average accuracy within each study catchment is reported in Table 1. In the framework presented in this paper, the local persistency is calculated from empirical observations. Given that node persistency dictates the fraction of time for which a node is active, one could expect that a model in which each node is randomly activated/deactivated through time in a way that is consistent with the local persistency could have a positive accuracy. In fact, the accuracy of such a random model for a node with persistency P_i is $P_i^2 + (1 - P_i)^2$, leading to a minimum accuracy of 50%. The accuracy of the hierarchical model was thus compared to the accuracy of the random model for all the study catchments. Figure S6 show the maps of local accuracy that would be obtained from the random model. Figure S4 shows that the mean accuracy of the hierarchical model (99$\pm$1.7%) is substantially higher than the accurcay of the random model (79 ± 7.5%).

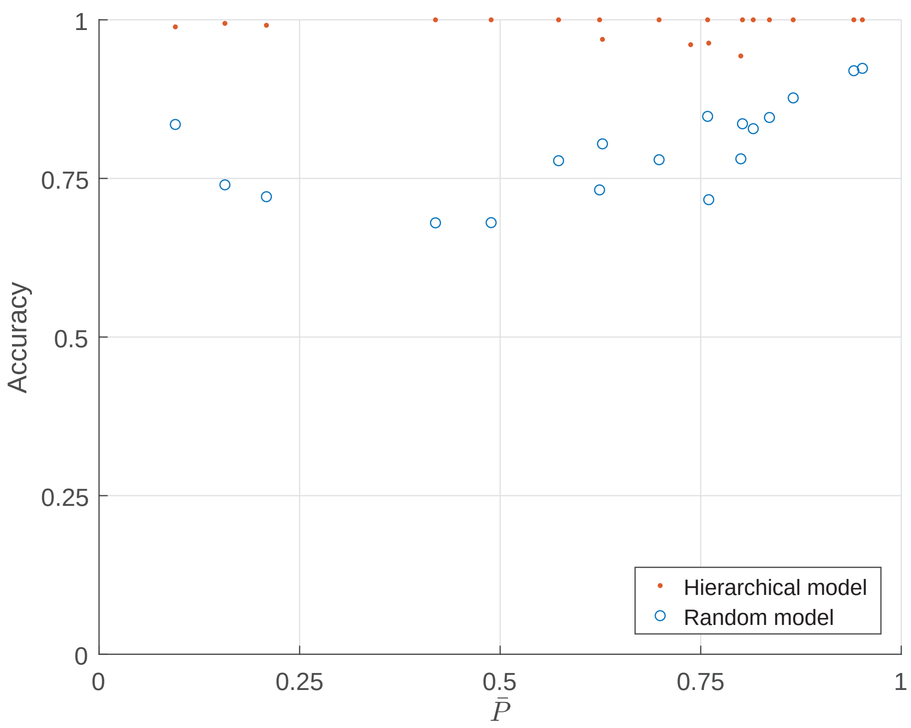

Figure S4: Accuracy of the hierarchical and random models as a function of the mean network persistency.

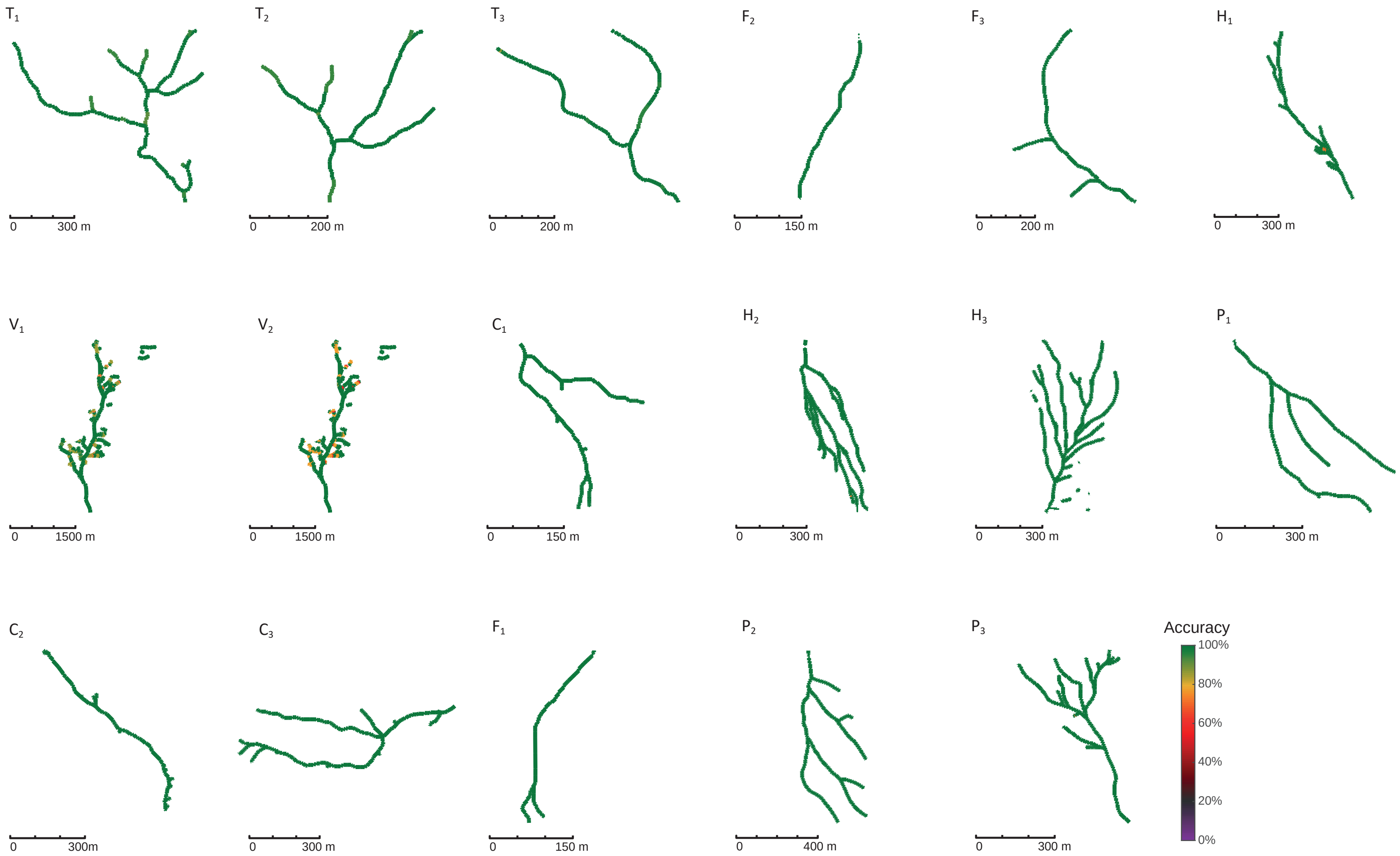

Figure S5: Maps of local accuracy of the hierarchical model for a set of the study catchments.

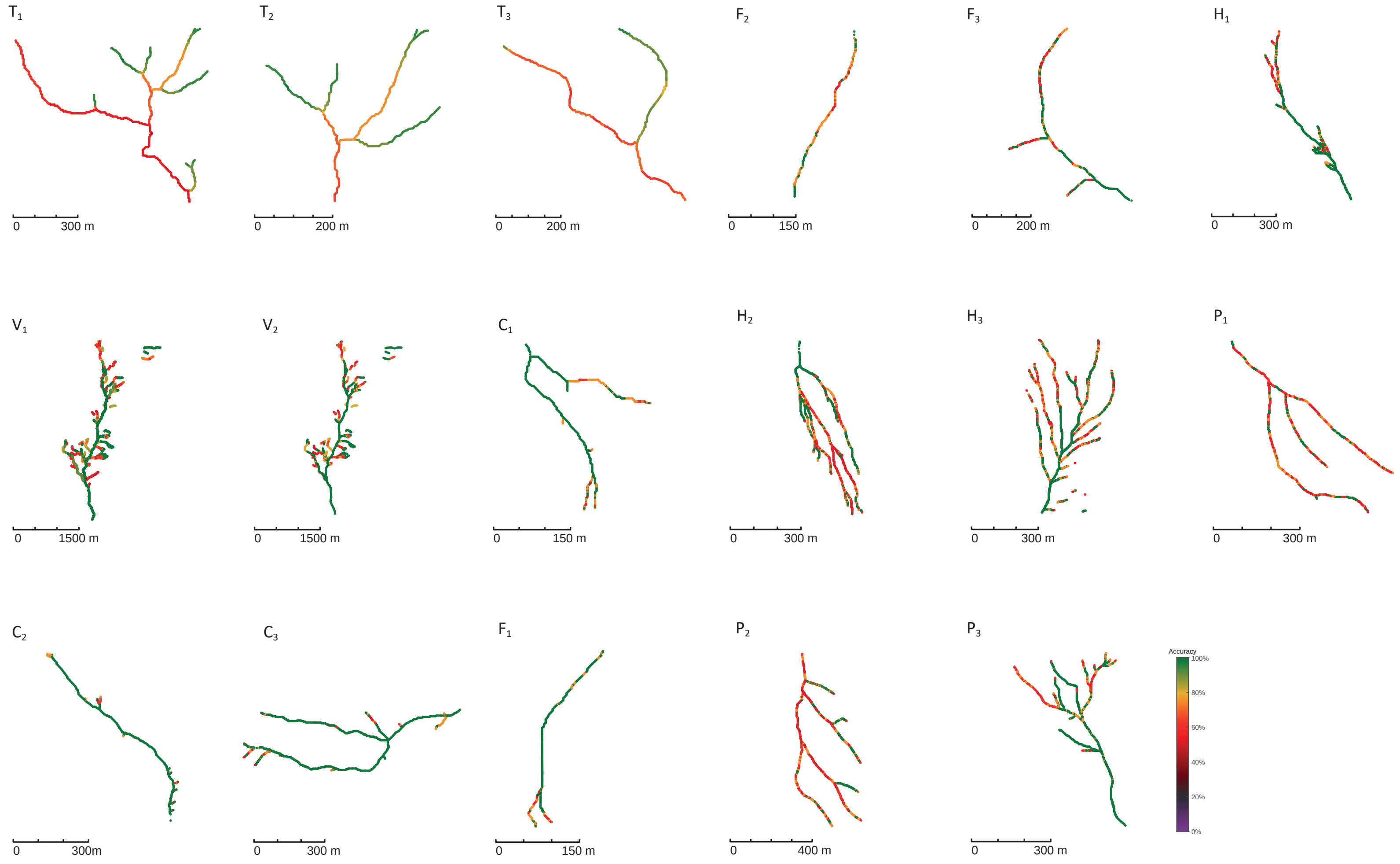

Figure S6: Maps of local accuracy of the random model.

1.6 Drainage density statistics

The data underlying Figures 2 and 3 of the main text are reported in Table S4, which reports the relevant drainage density statistics for the study catchments. The table also provides information on how the catchments have been grouped in the box and whiskers plots of Figure S3.

Table S4: Grouping of the study catchments and drainage density statistics

Group	Catchment code	$\bar{P}$	Area $[km^2]$	D_g $[km^{-1}]$	$\bar{D}$ $[km^{-1}]$	$dev(D)$ $[km^{-1}]$	$\bar{D}/D_g$	$dev(D)/D_g$	Mean precipitation $[mm/month]$	Mean ET $[mm/month]$
1	T2	0.10	0.23	5.62	0.52	1.18	0.09	0.21	99.4	114.2
	T3	0.16	0.46	2.85	0.45	0.85	0.16	0.30	99.4	114.2
	T1	0.21	0.63	4.71	0.97	1.37	0.21	0.29	99.4	114.2
2	P2	0.42	0.35	6.73	2.23	1.98	0.33	0.29	113.3	41.9
	P1	0.49	0.25	7.22	2.99	2.04	0.41	0.28	102.2	37.3
	F2	0.57	0.16	2.73	1.40	0.58	0.51	0.21	154.7	83.3
	H2	0.62	0.20	14.55	6.74	3.06	0.46	0.21	205.9	119.0
	V1	0.63	5.27	2.87	1.36	0.41	0.47	0.14	149.0	22.4
3	F3	0.70	0.37	2.92	1.62	0.45	0.55	0.15	155.1	83.3
	V2	0.74	5.27	2.87	1.59	0.31	0.56	0.11	172.4	25.9
	P3	0.76	0.73	2.88	1.46	0.35	0.51	0.12	NA	NA
	H1	0.80	0.14	12.54	8.62	1.51	0.69	0.12	198.5	73.0
	H3	0.82	0.43	8.88	6.46	1.55	0.73	0.17	197.9	118.8
	F1	0.84	0.14	3.11	2.49	0.26	0.80	0.08	140.1	71.7
4	C1	0.87	0.12	6.29	5.22	0.56	0.83	0.09	242.4	83.8
	C3	0.94	0.40	5.55	4.78	0.23	0.86	0.04	240.0	69.1
	C2	0.95	0.33	3.64	3.24	0.14	0.89	0.04	246.3	62.2

1.7 Correlation between mean network persistency and catchment area

Our analyses indicated that mean network persistency and catchment area are poorly correlated, with a correlation coefficient of 0.06. The scatter plot of $\bar{P}$ vs. A is shown in Figure S7.

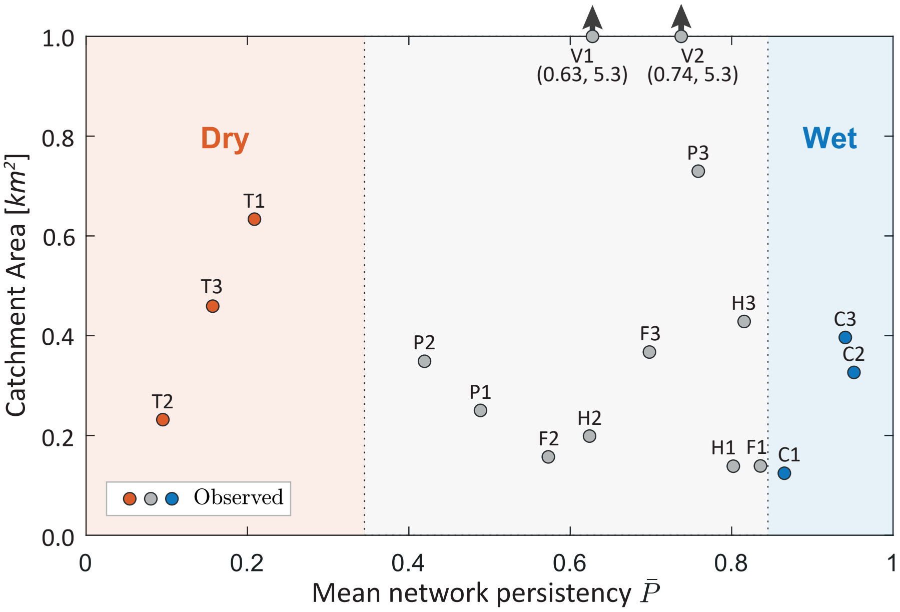

Figure S7: Scatter plot of catchment area and mean network persistency $\bar{P}$. The correlation coefficient is 0.06.

1.8 Analytical derivations

Average drainage density

A dynamical stream network is here described by a set of N nodes. Each node identifies the hydrological status (active vs. dry) of a suitably identified portion of stream network with length Δl_i. At any given time, the node i takes a binary status X_i (0 = dry / 1 = active). Therefore, at any time the active steam length can be calculated as the sum of the lengths associated to each active node,

$$L = \sum_{i=1}^{N} X_i \Delta l_i \ . \tag{S1}$$

The local persistency of node i, P_i, defined as the probability for the node i to be observed as active, can be calculated as the temporal average of the observed state of the node exploiting a series of field surveys of the active network (i.e. the average of X_i, sampled at different times).

Likewise, the average stream length can also be calculated as:

$$\bar{L} = \overline{\sum_{i=1}^{N} X_i \Delta l_i} = \sum_{i=1}^{N} \overline{X_i} \Delta l_i = \sum_{i=1}^{N} P_i \Delta l_i = \bar{P} L_g \tag{S2}$$

where $\bar{P}$ is the mean network persistency, $L_g = \sum_{i=1}^{N} \Delta l_i$ is the geomorphic length (corresponding to the length of the network when all the nodes are active), and the overline denotes time averaging. Equation (S2) shows that the definition of the maximum length of the network is a crucial step for the calculation of the mean persistency: in fact, for a given mean network length, the mean persistency is inversely proportional to L_{max}. Equation (1) of the main text is obtained by dividing both sides of equation (S2) by the catchment area.

General expression for the coefficient of variation of the active drainage density

The local status of each node, X_i, can be seen as a Bernoulli binary random variable. Accordingly, the local persistency is the expected value of X_i, while the variance of X_i can be expressed as: $Var(X_i) = P_i(1 - P_i)$. Let us now consider a generic couple of nodes i and j with local persistencies P_i and P_j, respectively. One can identify four joint probabilities of the states of these nodes as follows (*66*):

- $P_{11}^{ij} = Prob[X_i = 1 \; and \; X_j = 1]$
- $P_{00}^{ij} = Prob[X_i = 0 \; and \; X_j = 0] = 1 - P_j - P_i + P_{11}^{ij}$
- $P_{10}^{ij} = Prob[X_i = 1 \; and \; X_j = 0] = P_i - P_{11}^{ij}$
- $P_{01}^{ij} = Prob[X_i = 0 \; and \; X_j = 1] = P_j - P_{11}^{ij}$ (S3)

The above joint probabilities obey to three constraints: $P_{11}^{ij} + P_{10}^{ij} = P_i$, $P_{01}^{ij} + P_{11}^{ij} = P_j$ and $P_{11}^{ij} + P_{10}^{ij} + P_{01}^{ij} + P_{00}^{ij} = 1$. Incorporating these constraints into equation (S3) allows for the calculation of all the joint probabilities based on the local persistencies (P_i and P_j) and one joint probability (e.g. P_{11}^{ij}). In the general case, the covariance of the states of two nodes can be written as:

$$\begin{aligned} Cov(X_i, X_j) &= (1 - P_i)(1 - P_j)P_{11}^{ij} + (1 - P_i)(0 - P_j)P_{10}^{ij} + \\ &\quad (0 - P_i)(1 - P_j)P_{01}^{ij} + (0 - P_i)(0 - P_j)P_{00}^{ij} \\ &= P_{11}^{ij} - P_i P_j \end{aligned} \tag{S4}$$

Due to the symmetry of the covariance ($Cov(X_i, X_j) = Cov(X_j, X_i)$), there is no loss of generality in assuming $P_i \geq P_j$. Introducing the hypothesis of hierarchical activation of the nodes (*66*) is equivalent to imposing $P_{01}^{ij} = 0$ (i.e. the less persistent node j cannot be active if the more persistent node i is dry), or $P_{11}^{ij} = P_j$. As a consequence, the covariance between the states of two nodes reads:

$$Cov(X_i, X_j) = P_j(1 - P_i)\,. \tag{S5}$$

This expression is only dependent on the persistencies of the two nodes. Let us now consider a stream network with N nodes numbered in a decreasing

order of persistency (i.e. $P_i \geq P_j$ for $i < j$). Note that node numbering does not necessarily reflect the actual position of the nodes in the physical space. The states of the nodes can be represented in a vector of random variables $\boldsymbol{X} = (X_1, X_2, ..., X_N)$. The covariance between each possible couple of nodes can be summarized in the covariance matrix, the elements of which can be expressed as:

$$Cov(\boldsymbol{X}) = \begin{cases} P_i(1-P_i) & \text{for } j = i \\ P_i(1-P_j) & \text{for } j < i \\ P_j(1-P_i) & \text{for } i > j \end{cases} \tag{S6}$$

where i and j represent the row and column indexes, respectively. At any given time, the active stream length L is a linear combination of the states of all the nodes, as per equation (S1). The stream length variance is therefore expressed as the sum of all the elements of the covariance matrix, properly weighted by the Δl_i associated to each node:

$$Var(L) = \sum_{i=1}^{N}\sum_{j=1}^{N} cov(X_i, X_j)\Delta l_i \Delta l_j \, . \tag{S7}$$

Without loss of generality, to simplify the notation a constant unit length $\Delta l_i = \Delta l = 1$ is assumed for each node. All the relevant equations, however, can be easily generalized to the case $\Delta l_i \neq const$ by properly weighting each term associated to the node i (i.e. the status X_i or the local persistency P_i) with the corresponding Δl_i. First, let us express each local persistency as the sum of the mean network persitency, $\bar{P}$, and a local deviation from the mean, ΔP_i (i.e. $P_i = \bar{P} + \Delta P_i$). Note that $\sum_{i=1}^{N} \Delta P_i = 0$ by definition. Combining equations (S6) and S7, and using the above definitions, the stream length variance can then be written as:

$$\begin{aligned} Var(L) &= \left[\sum_{i=1}^{N} P_i(1-P_i)\right] + \left[2\sum_{i=2}^{N}\sum_{j=1}^{i-1} P_i(1-P_j)\right] \\ &= \left[N\bar{P}(1-\bar{P}) - \sum_{i=1}^{N}\Delta P_i^2\right] + \left[(N-1)N\bar{P}(1-\bar{P}) + 2\sum_{i=1}^{N} i\Delta P_i - 2\sum_{i=2}^{N}\sum_{j=1}^{i-1}\Delta P_i \Delta P_j\right] \\ &= N^2\bar{P}(1-\bar{P}) - \sum_{i=1}^{N}\Delta P_i^2 - 2\sum_{i=2}^{N}\sum_{j=1}^{i-1}\Delta P_i\Delta P_j + 2\sum_{i=1}^{N} i\Delta P_i \\ &\approx N^2\bar{P}(1-\bar{P}) + N^2\widetilde{\Delta P} \end{aligned} \tag{S8}$$

where the term $\widetilde{\Delta P} = 2/N^2 \cdot \sum_{i=1}^{N} i\Delta P_i$ represents a weighted spatial average of the persistency deviations around the mean. The weights associated to each

ΔP_i increases as i increases (i.e., as P_i decreases). Therefore, a bigger weight is attributed to the negative values of ΔP_i, and $\widetilde{\Delta P} \leq 0$. It is important to note that the last row of equation (S8) represents an approximation of the actual value of $Var(L)$, which is obtained from the full expression of $Var(L)$ by neglecting the following two terms: i) the term $\sum_{i=1}^{N} \Delta P_i^2$, that proves to be negligible when N is large (because it is of order N, while the other terms are of order N^2); ii) the term $2\sum_{i=2}^{N}\sum_{j=1}^{i-1} \Delta P_i \Delta P_j$, that is negligible because of the compensating effects originated by the interplay between the factors ΔP_i and ΔP_j when the summation indices vary. A detailed set of numerical simulations based on synthetic networks and available empirical data confirmed that the neglected terms are at least four orders of magnitude smaller then all the other terms of the equation. Equation (S8) was then combined with equation (S2) to derive equation (2) in the main paper.

Upper limits for the coefficient of variation and the standard deviation of the active drainage density

For a given stream network (i.e. a set of nodes with known local persistencies), the length variance is maximized if the activation/deactivation of the nodes follows a hierarchical order. In fact, the hierarchical activation scheme (for which $P_{01}^{ij} = 0$ if $i < j$) maximizes the joint probability that two arbitrary nodes are concurrently active ($P_{11}^{ij} = P_j$), as per equation (S3). This circumstance in turn maximizes the covariance of the states of each couple of nodes in the network (equation (S5)) and the active length variance (as per equation (S7)).

Furthermore, a stream network in which all the nodes share the same persistency originate an higher active length variance with respect to any other possible network with the same mean network persistency $\bar{P}$. Intuitively, this property is directly implied by the hierarchical activation scheme, according to which all the nodes are activated or deactivated at the same time as long as they share the same persistency. In such a case, the active length can only assume the limiting values of 0 and L_g, thereby ensuring the maximization of the temporal variance of the active length. The same conclusion can be obtained analyzing the term $\widetilde{\Delta P} \leq 0$ in equation (S8). $\widetilde{\Delta P}$, in fact, is a weighted spatial average of the fluctuations of persistency around the mean. In the case where all nodes share the same persistency, $\Delta P_i = 0$ for each i, and $\widetilde{\Delta P} = 0$, allowing the maximization of the length variance (as per equation (S8)). Based on equation (S8), the upper limit for the variance of the active stream length can be written as:

$$Var^{max}(L) \approx N^2 \bar{P}(1-\bar{P}) \tag{S9}$$

This equation can be easily combined with equation (S2) to obtain equation (3) in the main paper. Equation (S9) shows that the universal maximum variance of the active length is obtained when $\bar{P} = 0.5$, because under these circumstances the term $\bar{P}(1-\bar{P})$ is maximized. In such a case, $Var^{max}(L) = 0.25N^2$, where N is the geomorphic length due to the assumption that $\Delta l_i = \Delta l = 1$.

The universal upper bound of the length standard deviation is then:

$$dev(L) = \left[Var^{max}(L)\right]^{0.5} = 0.5L_g \tag{S10}$$

as reported in the main text.

Coefficient of variation of the drainage density for beta-distributed persistencies

The available empirical data show that the frequency distribution of the local persistency of the different nodes in the network, here denoted as $pdf(P)$, can be reasonably described by a Beta distribution with the first shape parameter set to 1:

$$pdf(P) = \frac{(1-P)^{\beta-1}}{B(1,\beta)} \,. \tag{S11}$$

In the above equation P is the local persistency, β is the second shape parameter of the distribution and $B(1,\beta) = \frac{1}{\beta}$. For each case study, the parameter β was calculated using the method of moments based on the mean network persistency, $\bar{P}$. In particular, the analytical expression of the first moment of the Beta distribution was exploited to calculate the free shape parameter as a function of the mean network persistency:

$$\beta = \frac{1-\bar{P}}{\bar{P}} \,. \tag{S12}$$

The analogous expression for the second-order moment of the Beta distribution can be used to evaluate the spatial variance of the local persistency as:

$$Var(P) = \frac{\bar{P}^2(1-\bar{P})}{1+\bar{P}} \tag{S13}$$

Likewise, $\widetilde{\Delta P}$ can be expressed as follows:

$$\widetilde{\Delta P} = -\frac{\bar{P}(1-\bar{P})}{2-\bar{P}} \tag{S14}$$

Equations (S8) and (S14) were then combined to obtain the following expression of the variance of the active length when the local persistencies are beta-distributed:

$$Var(L) = N^2 \frac{\bar{P}(1-\bar{P})^2}{1-\bar{P}} \tag{S15}$$

Equation (4) of the main paper is then derived combining equations (S2) and (S15).

1.9 Monitoring hierarchical networks

If the activation and deactivation of the nodes of a dynamic stream network obeys to a purely hierarchical scheme, the monitoring of active stream length dynamics can be significantly facilitated. In fact, the hierarchical assumption can be used to deduce the status of some nodes in the network from the observed status of other nodes. Specifically, if a node is observed as wet then all the nodes in the network with a larger persistency should be wet. Likewise, if a node is dry, then all the nodes with a smaller persistency should be dry as well. One might argue that prior to the field campaign for the monitoring of active stream network dynamics the persistencies of the nodes are not known. However, as long as the surveys take place, this hierarchy is progressively revealed and, crucially, the hierarchies emerging during any survey will remain unchanged for the entire campaign. Thus, as the number of available active stream network maps increases, the ability to extrapolate in space information about the status of the nodes increases as well. To quantify the number of nodes that do not need to be visited during a field campaign (because their status can be deduced from the status of other nodes), a series of numerical simulations was performed in which we simulated the dynamics of the stream network by simulating a hierarchical chain of nodes and the position of the transition between wet and dry nodes in the chain. Likewise, the number of nodes that has to be visited using the information available prior to the survey is computed. These numerical simulations consisted in the following steps: i) a sequence of N nodes with a dichotomic status and a fixed distribution of the local persistencies is considered; ii) the nodes are first re-ordered in a chain following a decreasing order of local persistency (the first node is the most persistent, the last node the least persistent); iii) an auxiliary counting variable, k, is set equal to 1; iii) the position of the unique transition between wet and dry nodes in the chain is identified by selecting randomly one of the existing groups of nodes in the chain; if $k = 1$ the position of the transition is set within the unique group containing all the N nodes of the chain; iv) the selected group is then randomly split into two sub groups (namely, the group of wet nodes and the group of dry nodes) and a new group is thus created; v) a new hierarchy is created between these two node subgroups by assigning an apparent persistency P_a to the existing $k+1$ groups as follows: $P_a = s/k$, where s is the number of times that a group of nodes was observed as active during the campaign up to the kth survey; vi) the number of nodes to be visited during the kth survey to characterize the whole active network is then computed as the sum of the number of nodes that need to be surveyed to identify the groups within which the transition takes place (i.e., the number of existing groups k) plus the number of nodes that need to be visited to identify the precise location of that transition within the nodes of the relevant groups (all the nodes that are included in the two groups where the transition takes place); vii) the counting variable k is increased to the subsequent integer number and the procedure starts again from step iii). The procedure stops when a pre-determined number of field surveys (say, K) is performed. The percentage of nodes that has to be surveyed is then calculated as the number of surveyed

nodes during the whole campaign scaled to the number of nodes that potentially have to be visited (i.e., $N\,K$). The simulations were performed with different combinations of K, N and $\bar{P}$, and the results are shown in Figure S8. The plot indicates that, for hierarchical networks, the number of nodes to be visited is between 60 to 30 % of the overall number of nodes that should be surveyed in non-hierarchal networks, with smaller percentages associated to field campaigns made up of a larger number of surveys.

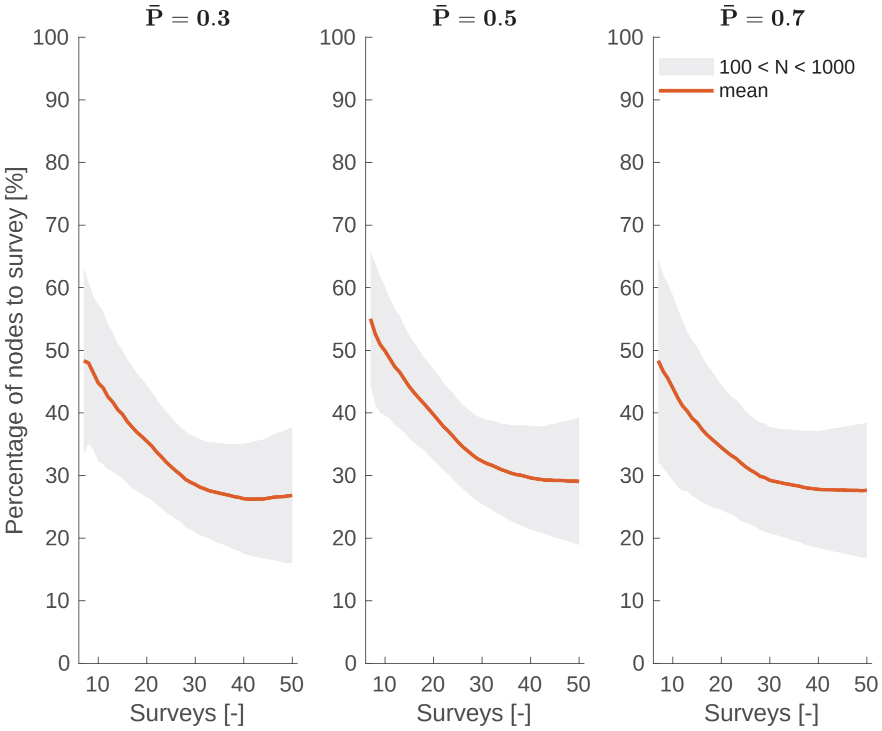

Figure S8: Percentage of nodes to survey in perfectly hierarchical networks, as a function of the number of performed surveys and the number of nodes, N. The orange line shows the average, while the shadowed area shows the range obtained by varying the number of nodes in the network between 100 and 1000.

References

58. L. Pfister, N. Martìnez-Carreras, C. Hissler, J. Klaus, G. E. Carrer, M. K. Stewart, and J. J. McDonnell, "Bedrock geology controls on catchment storage, mixing, and release: a comparative analysis of 16 nested catchments," *Hydrological Processes*, vol. 31, pp. 1828–1845, 2017.

59. O. national des étiages, “Onde dataset,” 2020. Fata retrieved from https://onde.eaufrance.fr/.

60. “Hubbard brook experimental forest: Total daily precipitation by watershed, 1956 - present.” Available at https://doi.org/10.6073/pasta/c64ad38eef4f56d9e34749f166f64caa.

61. P. J. Edwards and F. Wood, “Fernow experimental forest daily precipitation.” Available at https://doi.org/10.2737/RDS-2011-0014.

62. C. F. Miniat, S. H. Laseter, W. T. Swank, and L. W. J. Swift, “Daily precipitation data from recording rain gages (rrg) at coweeta hydrologic lab, north carolina.” Available at https://doi.org/10.2737/RDS-2017-0031.

63. “Usgs monthly et data.” https://earlywarning.usgs.gov/ssebop/modis/8-day/607.

64. G. Senay, “Satellite psychrometric formulation of the operational simplified surface energy balance (SSEBop) model for quantifying and mapping evapotranspiration,” *Applied Engineering in Agricolture*, vol. 34, pp. 555–566, 2018.

65. R. Allen, M. Smith, W. Pruitt, and L. Pereira, “Modification of the FAO crop coefficient approach,” *Proceedings of the International Conference*, pp. 124–132, 1996.

66. G. Botter and N. Durighetto, “The stream length duration curve: a tool for characterizing the time variability of the flowing stream length,” *Water Resources Research*, vol. 56, 2020.
